# Neutrophils in liver diseases: pathogenesis and therapeutic targets

**DOI:** 10.1038/s41423-020-00560-0

**Published:** 2020-11-06

**Authors:** Kai Liu, Fu-Sheng Wang, Ruonan Xu

**Affiliations:** 1 Peking University 302 Clinical Medical School, Beijing, China; 2 Treatment and Research Center for Infectious Diseases, The Fifth Medical Center of PLA General Hospital, National Clinical Research Center for Infectious Diseases, Beijing, China

**Keywords:** Liver disease, Liver injury, Neutrophil, Innate immunity, Immunotherapy, Neutrophils, Mechanisms of disease

## Abstract

Previously, it was assumed that peripheral neutrophils are a homogeneous population that displays antimicrobial functions. However, recent data have revealed that neutrophils are heterogeneous and are additionally involved in tissue damage and immune regulation. The phenotypic and functional plasticity of neutrophils has been identified in patients with cancer, inflammatory disorders, infections, and other diseases. Currently, neutrophils, with their autocrine, paracrine, and immune modulation functions, have been shown to be involved in liver diseases, including viral hepatitis, nonalcoholic steatohepatitis, alcoholic liver disease, liver fibrosis, cirrhosis, liver failure, and liver cancer. Accordingly, this review summarizes the role of neutrophils in liver diseases.

## Introduction

Neutrophils are polymorphonuclear cells that are abundant in peripheral blood and participate in the antimicrobial response and inflammation-induced tissue damage in vivo. The phenotypic and functional plasticity of neutrophils complicates the etiology of liver diseases, from chronic hepatitis to liver cirrhosis and hepatocellular carcinoma (HCC). During liver injury, neutrophils can be recruited rapidly and perform a series of inherent antibacterial functions, including degranulation, reactive oxygen species (ROS) generation, phagocytosis, and the formation of neutrophil extracellular traps (NETs). Meanwhile, neutrophils are also involved in the regulation of the adaptive immune response through direct contact or the production of indirect mediators.^1^ The multiple functions of neutrophils make them important in the prevention of microbial invasion, tissue injury or repair, regulation of innate and adaptive immunity, and promotion of cellular transformation in liver diseases. Therefore, neutrophils are also considered to act as a “double-edged sword”. This review summarizes the current progress and some critical issues of neutrophils in liver diseases.

## The inherent role of neutrophils

Neutrophils are the most abundant leukocytes in peripheral circulation and are well characterized in the host defense against bacteria, fungi, and viruses, even playing a significant role in shaping adaptive immunity.^2^ Generally, neutrophils have a half-life shorter than one day; however, their half-life can be lengthened to more than 5 days in certain tissues and under certain specific conditions,^3^ which provides sufficient time for the formation of neutrophil plasticity under a special microenvironment. Granulocyte colony-stimulating factor (G-CSF) is regarded as the major activator of neutrophil mobilization and survival.^4^ Infiltration of neutrophils is very common in liver tissue. The damage- and pathogen-associated molecular patterns (DAMPs and PAMPs) released from aseptic inflammatory or infected tissue, respectively, are the principal activators of neutrophils. Neutrophil surface receptors (P-selectin glycoprotein ligand 1, L-selectin, CD44, and macrophage antigen 1 (Mac-1)) mediate the adhesion and migration of neutrophils through their interaction with adhesion molecules (E-selectin and P-selectin) and the integrin superfamily (intercellular adhesion molecules (ICAMs) and vascular cell adhesion molecules (VCAMs)) expressed on endothelial cells. The migration speed, polarity, and direction of neutrophils are also influenced by other chemoattractants (e.g., complement 5a (C5a), interleukin (IL)-8, and IL-6). Simultaneously, neutrophils are also capable of directly activating Kupffer cells and endothelial cells, resulting in the upregulation of adhesion molecules (ICAM-1 and VCAM-1) and the promotion of cell adhesion.^5^ Dipeptidase-1, a major adhesion receptor on hepatic endothelial cells, also mediates neutrophil adhesion in an enzyme activity-independent manner.^6^ Apoptotic neutrophils are eliminated mainly by macrophages, which contributes to localized tissue inflammation. Meanwhile, activated macrophages secrete both G-CSF and granulocyte-macrophage colony-stimulating factor (GM-CSF) to slow neutrophil apoptosis.^7^ Neutrophil necrosis leads to the release of harmful proinflammatory chemokines, cytokines, and granule proteins, which might further aggravate local tissue damage.^8^ Neutrophil necroptosis enhances macrophage efferocytosis through the expression of “eat me signals”.^9^ Meanwhile, cytokine-activated macrophages are needed to eliminate excess neutrophils and promote inflammatory remission.^10^

At local damaged sites, neutrophils capture pathogens by direct interaction or through Fc receptors, integrin, and complement receptors. The antibacterial reactions are mainly mediated by phagocytosis and degranulation-containing elastase, lysozyme, myeloperoxidase (MPO), and other defensins. Simultaneously, superoxide anions and ROS are produced during the progression of the respiratory burst.^11^ Recently, extracellular fibrous structures, named NETs, which include a large amount of extruded DNA, cationic proteins (elastase, cathepsin G, lactoferrin, MPO, and neutrophil gelatinase-associated lipoprotein (NGAL)) from the cytoplasm and granules, have been demonstrated to have the capacity to capture and kill pathogenic microorganisms. NETs carrying NGAL released by neutrophils are regarded as important mediators in the defense against bacterial infection in vivo.^12^

The defense activities of neutrophils are harmoniously coordinated and regulated, leading to rapid bacterial clearance, inflammation regression, and tissue repair. The interaction of neutrophils with macrophages and other types of cells determines liver immune homeostasis and tissue repair. During viral hepatitis, alcoholic liver disease, metabolic injury, and other conditions, persistent infiltration and multiple dysfunctions of neutrophils usually aggravate liver injury. We will elaborate on this in detail later.

## The role of neutrophils in viral hepatitis

A lack of sufficient liver biopsy samples and suitable animal models means that the role of the innate immune system, especially neutrophils, in acute and chronic viral hepatitis is not fully understood. Intrahepatic gene expression profiles show that multiple innate immunity-related genes are inhibited during chronic hepatitis B infection, independent of HBV replication, suggesting that the innate immune response is seriously impaired in patients with chronic HBV infection.^13^ NETs released by neutrophils are regarded as the main executors of anti-infective and antiviral immunity.^14,15^ In chronic hepatitis B infection, the amount of NETs decreased and correlated negatively with HBsAg, HBeAg, and HBcAb levels. HBV C protein and HBV E protein might inhibit the release of NETs by inhibiting ROS generation and autophagy, ultimately leading to the delayed elimination of HBV virions.^16^ In a mouse hepatitis virus-A59 model, excessive NET release by neutrophils was associated with fulminant hepatitis.^17^ In patients with HBV infection, C-X-C motif chemokine ligand 8 (CXCL8) produced by T cells is the major attractant for neutrophil influx into the liver, which increases the expression of triggering receptor expressed on myeloid cells 1 (TREM1) and aggravates the neutrophil-mediated immunopathology of the liver.^18^ Meanwhile, the higher neutrophil-to-lymphocyte ratio (NLR) at the 24th week after the onset of interferon treatment predicted the elimination of the replicative HCV-RNA strand in the liver during the antiviral treatment of patients with chronic hepatitis C.^19^

In a viral hepatitis model induced by lymphocytic choriomeningitis virus infection, deletion of neutrophils increased the viral load, aggravated the T-cell response, and increased liver injury, while IL-33 treatment contributed to reducing liver inflammation by promoting the aggregation and immunosuppressive phenotype transformation of neutrophils within the liver.^20^ In acute murine viral hepatitis, increased infiltration of neutrophils into liver tissue is also exacerbated by endogenous IL-33 deficiency.^21^ These data indicated that IL-33 might be a target for reducing neutrophil infiltration in hepatitis, and IL-33 targeting therapy might benefit the outcome of viral hepatitis.

## The role of neutrophils in nonalcoholic fatty liver disease

Nonalcoholic fatty liver disease (NAFLD) represents a spectrum of diseases, ranging from simple steatosis to nonalcoholic steatohepatitis (NASH), liver cirrhosis, and even liver cancer. Preexisting diseases, including obesity, type 2 diabetes, hyperlipidemia, and insulin resistance, are regarded as the major risk factors for the development of NAFLD.^22^ Hepatic steatosis is a common consequence of hepatic injury in response to alcohol, toxins, chemotherapy, or metabolic syndrome with insulin resistance and has become the most common liver disease worldwide, including in China.^23–25^ Hepatic steatosis can progress to NASH, which is characterized by inflammation and metabolic disorders. Neutrophil infiltration is associated with liver inflammation and subsequent inflammation-induced pathological injury, which is often caused by dietary carbohydrates and cholesterol and is related to the severity of NASH.^22^

Human NASH is characterized by hepatic neutrophil infiltration and upregulation of the major neutrophil-recruiting chemokines (CXCL1 and IL-8) in human hepatocytes. However, mice fed a high-fat diet (HFD) only develop a fatty liver without progression to NASH, in which the disparity of p38α (also known as mitogen-activated protein kinase 14 (MAPK14)) activation is an important determinant. Human NASH features strong p38α activation, which induces caspase 3 (CASP3) cleavage, AF4/FMR2 family member 4 (AFF4; also known as CHOP) expression, and BCL2 apoptosis regulator (BCL2) phosphorylation, thereby exacerbating hepatocyte death. However, mice fed a HFD only showed weak p38α activation, which upregulated genes involved in fatty acid β-oxidation that may act to compensate for hepatocyte fat accumulation.^26,27^ In patients with NASH, serum NET levels are markedly increased. In an animal model of NASH, free fatty acids also stimulated the formation of NETs in vivo and in vitro. Inhibition of NET formation did not affect steatosis but decreased inflammatory cell infiltration and the production of inflammatory cytokines, even slowing down the progression of NASH to liver cancer.^28^ Serum neutrophil-derived lipocalin 2 is also significantly upregulated in patients with NASH, which triggers C-X-C motif chemokine receptor 2 (CXCR2) expression in an autocrine manner, ultimately promoting neutrophil and macrophage infiltration and aggravating liver inflammation and hepatocyte injury.^29^ In addition, high neutrophil elastase and low α-1 antitrypsin activities predict the severity of experimental NASH.^30^ Neutrophils can directly kill hepatocytes by secreting MPO to enhance oxidative stress; indeed, the concentration of MPO in patients with NASH is higher than that in patients with simple steatosis.^31^ At the same time, MPO can activate hepatic stellate cells and promote liver fibrosis.^32^ Antibodies against neutrophils improved metabolic syndrome and reduced functional disorders and lipid deposition.^33^

In short, hepatocyte injury is caused by metabolism and induced by neutrophil infiltration and activation, which in turn mediates hepatocyte injury through ROS, inflammatory mediators, and NETs.^34^ However, neutrophils do not always play a negative role. High expression of microRNA-223 (miR-223) by neutrophils has immune regulatory functions in a variety of liver diseases. Deletion of miR-223 in a mouse model induced a full range of NAFLD, from simple fatty liver to HCC, combined with increased inflammation.^35^

## The role of neutrophils in alcoholic liver disease

**Fig. 1:**
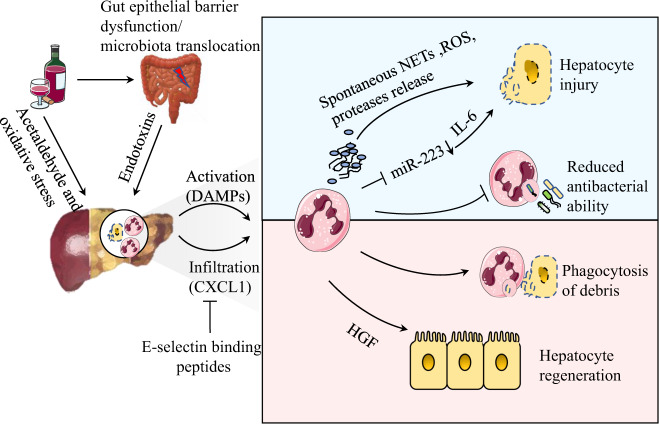
Multiple roles of neutrophils in the microenvironment of alcoholic liver disease. Alcohol causes liver damage via acetaldehyde/oxidative stress and endotoxins produced during microbiota translocation. DAMPs and chemokines activate and recruit neutrophils in different ways. On the one hand, hepatocyte injury can be induced by excessive NETs and ROS, and decreased miR-223 promotes IL-6 production, which further contributes to liver injury. The decreased antibacterial ability aggravates infection susceptibility and mortality in advanced liver diseases. On the other hand, neutrophils inhibit liver injury by phagocytosing necrotic cellular debris and secrete hepatocyte growth factor (HGF) to assist hepatocyte regeneration

Alcoholic liver disease (ALD) includes a series of disorders from simple fatty liver to alcoholic hepatitis, which can also progress to liver cirrhosis and liver cancer without proper treatment. The pathology of alcohol-related liver injury is very complicated, including the simultaneous occurrence of direct alcohol-related liver injury, dysfunction of the immune inflammatory response to intestinal injury, and microbial translocation. In particular, the unbalanced intestinal bacteria and increased permeability of the gastrointestinal mucosa eventually activate an inflammatory response, induce aseptic necrosis and apoptosis of hepatocytes, and promote neutrophil infiltration, ultimately triggering a series of pathophysiological changes (Fig. 1).^36^

It is generally accepted that significant neutrophil infiltration into the liver after long-term drinking can also be promoted by drinking heavily over a short period of time. Acute alcohol intake leads to a neutrophil immune imbalance that results in the spontaneous release of NETs. However, the formation of NETs by these neutrophils is decreased upon secondary antigen stimulation and might lead to weakening of the antibacterial ability. Simultaneously, the scavenging effect of macrophages on NETs decreases, resulting in persistent liver inflammation and injury by NETs.^37^ Extensive oxidative modification of albumin in patients with severe alcoholic hepatitis further activates neutrophils, which induces persistent oxidative stress and inflammation in these patients.^38^ Blocking neutrophil infiltration using anti-CXCL1 treatments could significantly reduce alcohol-induced liver injury.^39^

miR-223 is the most abundant microRNA (miRNA) in neutrophils and plays an important role in the modulation of neutrophil function in ethanol-induced mouse liver injury. Elevated serum miR-223 levels were associated with increased infiltration of circulating neutrophils and high levels of alanine transaminase and aspartate transaminase. In addition, miR-223 inhibited IL-6 expression and subsequently inhibited p47^phox^ expression in neutrophils, which alleviated ethanol-induced hepatic injury and ROS production. However, in alcoholic patients, miR-223 in neutrophils was downregulated and IL-6 and p47^phox^ expression were higher than the levels in healthy controls.^40^ miR-223 could also restore liver inflammation and early fibrosis by inducing the conversion of proinflammatory macrophages to anti-inflammatory types.^41^ However, in the acetaminophen (APAP) injury model, mitochondrial DNA induces negative feedback activation of miR-223 through Toll-like receptor 9 (TLR-9), which limits the overactivation of neutrophils and liver injury, which might help to reveal the role of neutrophils in ALD.^42^ Some studies have shown that the combined use of programmed cell death 1 (PD-1) with T-cell immunoglobulin and mucin domain-containing protein 3 (TIM-3) inhibitors might partially restore the antibacterial ability of neutrophils in patients with alcoholic hepatitis.^43^ Furthermore, E-selectin binding peptides inhibited the supplementation of neutrophils to inflammatory sites and reduced the levels of proinflammatory factors and chemokines in an alcoholic liver model, which might be used to control liver inflammation.^44^

Although there have been many reports on the negative roles of neutrophils in alcoholic hepatitis, some clinical studies have drawn different conclusions. On the one hand, the cytokines and chemokines that promote neutrophil infiltration are related to the severity of alcoholic hepatitis by increasing ROS, proteases, and inflammatory mediators, which promote liver inflammation and fibrosis. On the other hand, neutrophils are able to clear necrotic hepatocytes and are an essential source of hepatocyte growth factor (HGF) in patients with severe alcoholic hepatitis, thus participating in hepatocyte regeneration in severe alcoholic hepatitis.^45^ Moreover, neutrophil dysfunction leads to infection susceptibility and increased mortality in advanced liver diseases. A histological screen identified that the degree of neutrophil infiltration is related to the recovery from acute alcoholic hepatitis and is related to 90-day mortality in patients with alcoholic hepatitis.^46^ Therefore, the balance of neutrophils between the antibacterial and anti-inflammatory processes needs to be carefully considered in patients with ALD.

## The role of neutrophils in liver fibrosis and cirrhosis

Under continuously uncontrolled inflammation and stimulation, apoptosis and pyroptosis of hepatocytes cause serious liver injury, liver fibrosis, or liver cirrhosis. Neutrophils are regarded as important participants that affect the process of liver injury and repair and increase the risk of infection and susceptibility to organ failure at the end stage of liver cirrhosis. During liver cirrhosis, high levels of proinflammatory cytokines, intestinal barrier damage, microflora translocation, portal shunt, circulatory system endotoxins, and the accumulation of other PAMPs contribute to the dysfunction of neutrophils. The overexpression of CD11b (also known as integrin subunit alpha M) and dysfunctional chemotaxis, phagocytosis, degranulation, ROS production, and NET production in neutrophils ultimately lead to deteriorated liver function.^47^ Simultaneously, stellate cells activated by IL-17+ neutrophils release transforming growth factor beta (TGF-β), resulting in type I collagen deposition and aggravation of fibrosis.^48^

However, neutrophils also have beneficial roles in liver fibrosis or cirrhosis. A deficiency in MPO release in decompensated alcoholic cirrhosis leads to a decreased antimicrobial ability of neutrophils.^45^ Meanwhile, neutrophils perform a beneficial function through their production of HGF^46^ or the promotion of early collagen degradation.^49^ Coculture of normal neutrophils with plasma from patients with liver cirrhosis caused phenotypic and functional changes similar to those observed in patients with liver cirrhosis, which indicated that the damage might be reversible.^50^ A retrospective study of patients with liver cirrhosis found that a high NLR was an independent predictor of hospitalization days and 90-day mortality, which was ultimately associated with higher 1-year mortality.^51^

In patients with decompensated liver cirrhosis, long-term and chronic activation of neutrophils, caused by large amounts of plasma inflammatory substances, leaves them in a state of exhausted fatigue in which neutrophils lose their ability to effectively deal with harmful external substances and show reduced overall anti-infection ability. The increased production of basic neutrophil ROS and deficiency of phagocytic function in these patients are associated with an increased risk of infection, organ dysfunction, and death.^47^ At present, infection is still one of the leading causes of death in patients with decompensated cirrhosis,^52^ and the bactericidal deficiency of neutrophils can be reversed by the activation of TLR7/8 in vitro.^53^ Therefore, both improvement in liver function and control of infection are necessary to improve the final outcomes of patients with liver cirrhosis.

## The role of neutrophils in liver failure

During the progression of liver cirrhosis, the immune function of the liver continues to deteriorate, accompanied by portal hypertension, intestinal barrier damage, and bacterial translocation, which might eventually lead to liver failure.^54^ Liver failure includes acute liver failure, chronic liver failure, subacute liver failure, and acute-on-chronic liver failure (ACLF), which are differentiated according to their pathological features and speed of disease progression.

Acute liver failure represents a unique syndrome of severe injury to liver cells accompanied by altered coagulation and mentation in the absence of chronic liver disease.^55^ A common cause of death in patients with acute liver failure is systemic complications, which follow the release of proinflammatory cytokines and DAMPs from necrotic hepatocytes, endothelial cells, and leukocytes. Liver transplantation provides a life-saving alternative, and the NLR can be used as a practical index of early graft dysfunction for living donor liver transplantation.^56^

Paracetamol abuse is the most commonly observed reason for acute liver failure in developed countries. In APAP-induced liver failure, depletion of neutrophils protects against APAP toxicity when given as a 24-h pretreatment.^57^ The increased blood ammonia contributes to hepatic encephalopathy and could cooperate with endogenous DNA and IL-8 to regulate the expression of TLR-9 on the surface of neutrophils, decrease phagocytic activity, and increase the spontaneous oxidative burst.^58^ The increased TLR-9 but decreased TLR4 expression on functionally exhausted neutrophils correlates with the degree of encephalopathy.^58^ Excessive activation of neutrophils during APAP-induced ALF could also increase the susceptibility to sepsis, which might contribute to the high mortality.^59^ Although the depletion of neutrophils in APAP-induced liver failure models reduces early hepatocyte necrosis, it also dampens hepatocyte regeneration by reducing the levels of matrix metalloproteinases (MMPs);^60^ therefore, the balance of neutrophils is important to maintain normal liver function.

ACLF is a syndrome that occurs under liver cirrhosis and is characterized by organ failure and a high mortality rate within 28 days.^61^ During the course of ACLF, both a proinflammatory state and an immunosuppressive state might occur at the same time in which systemic and proinflammatory reactions occur in the early stage and an immunosuppressive reaction follows in the later stage, which manifests as immune paralysis, lymphocytopenia, and secondary infection.^62^ The state of the immune response depends on the host and the intensity of the pathogens, while the majority of patients with ACLF with liver cirrhosis are in a state of systemic immune exhaustion because of long-term chronic liver disease and tend to lose their normal resistance to infection, which ultimately contributes to liver failure.^11^ In patients with ACLF, phenotypic and functional changes in neutrophils are generally associated with outcomes. The expression of CXCR1/2 on neutrophils was lower than that in patients with chronic hepatitis, which is linked to the dysfunction of neutrophils and the severity of the disease.^59^ Blocking CXCR1/2 reduces the production of inflammatory mediators and hepatocyte necrosis.^63^ NGAL is regarded as a biomarker of ACLF and correlates with liver failure and systemic inflammation.^64^

The Model for End-Stage Liver Disease (MELD) score is a powerful and effective index to predict the short-term survival of patients with end-stage liver disease. Moreover, the NLR can also be used as an objective parameter to predict prognosis, independent of the MELD and cirrhosis stage. Further studies should highlight the effect of the number and function of neutrophils to optimize the therapeutic regimen used to treat liver failure.^65^

For patients with HBV-associated ACLF, G-CSF treatment has demonstrated safety and efficacy, which contribute to increasing the liver CD34+ cell and peripheral neutrophil count, reducing the MELD score, avoiding septicemia and new infections, and finally improving overall survival in patients with the disease.^66–68^ Meanwhile, for patients with pediatric ACLF, G-CSF treatment did not demonstrate significant efficacy in improving the outcome^69^ because ACLF in children can rapidly develop into multiple organ failure. G-CSF is also used to treat advanced liver cirrhosis and severe alcoholic hepatitis in which increased CD34+ cells and neutrophils are also associated with its efficacy (Table 1).

**Table 1:** Clinical studies of G-CSF for the treatment of liver disease

PMID number	Clinical regimen	Types of diseases	Outcomes	Changes of neutrophil and hematopoietic stem cells
22119930	Experimental group: 5 μg/kg G-CSF subcutaneously (12 doses; *n* = 23) plus standard medical therapy control group: placebo (*n* = 24) plus standard medical therapy	ACLF	Increased 60-day survival; decreased CTP, MELD, SOFA scores and complications	Increased CD34+ cells in the liver tissue at day 30 and neutrophil count
25749502	Experimental group: subcutaneous G-CSF (5 μg/kg/day) for 5 days and then every third day (12 total doses), along with subcutaneous darbopoietin α (40 mcg/week) for 4 weeks (*n* = 29), control group: only placebos (*n* = 26)	Advanced cirrhosis	Increased 12-month survival; reduced liver severity scores and sepsis	A trend of increasing CD34+ cells
24935272	Experimental group: medical therapy plus G-CSF (*n* = 23) at a dose of 5 μg/kg subcutaneously every 12 h for 5 consecutive days control group: medical therapy only	Severe alcoholic hepatitis	Increased 90-day survival and the number of CD34+ cells: decreased CTP, MELD, and mDF scores at 1, 2, and 3 months	Increase in the number of neutrophil and CD34+ cells in peripheral blood on day 6
32405176	Experimental group: GCSF therapy at 5 mcg/kg/day for 5 days along with standard medical care (*n* = 15) control group: standard medical care only (*n* = 16)	Children with ACLF	Ineffective in improving the survival outcome on day 30 and 60	A trend of CD34+ cells increase on day 7; the granulocyte counts became similar by day 30 and 60
28840583	Experimental group: G-CSF (5 μg/kg/day, for 6 consecutive days) in addition to standard medical therapy with antiviral drugs control group: standard medical therapy with antiviral drugs	ACLF	Improved survival and clinical recovery; prevented renal failure and hyponatremia	Increased mean white blood cell (WBC) count at day 15 in absence of infection
23467275	Experimental group: G-CSF (5 μg/kg per day, six doses) treatment plus standard therapy (*n* = 27), control group: received standard therapy only (*n* = 28)	ACLF	Promoted CD34+ cell mobilization; improved Child-Turcotte-Pugh score on day 30 and survival rate at 3 months	Increased neutrophil and CD34+ cell counts from day 3 to day 15

## The role of neutrophils in liver cancer

**Fig. 2:**
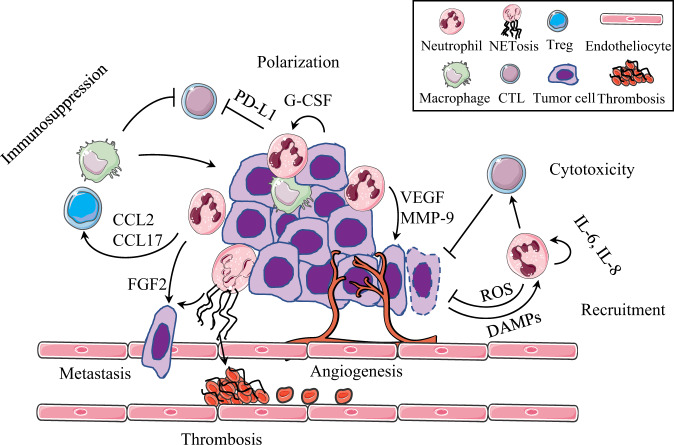
Multiple roles of neutrophils in the microenvironment of hepatocellular carcinoma. Neutrophils have the capacity to inhibit or promote tumor growth dependent on the tumor microenvironment. IL-6- and IL-8-recruited neutrophils kill tumor cells directly via ROS. Tumor cell-derived G-CSF polarizes neutrophils toward a tumorigenesis phenotype, which inhibits T-cell function in a cell–cell contact manner via PD-L1. Tumor-associated neutrophils (TANs) secrete VEGF and MMP-9 to induce angiogenesis, produce FGF2 to promote tumor cell metastasis, secrete CCL-2 and CCL-17 to recruit Treg cells and macrophages that exert immunosuppressive roles, release NETs to promote tumor-related thrombosis, and aggravate the body’s hypercoagulable state

Unlike other malignant tumors, HCC mainly occurs in a chronic inflammatory environment.^70^ Neutrophils are found in large quantities and with obvious heterogeneity in the microenvironment of liver cancer. The timely recruitment of neutrophils is controversial in anti-infection and tumorigenesis, showing different functional characteristics after being converted into tumor-associated neutrophils (TANs), which is summarized in Fig. 2. The TANs induced by type I interferon can be polarized to the antitumor N1 phenotype, which has the capacity to kill tumor cells and stimulate T-cell immunity. Meanwhile, the N2 phenotype induced by G-CSF can promote tumor development by inhibiting the T-cell response and upregulating angiogenic factors.^71^ There is a lack of specific cell surface markers; therefore, these tumor infiltrated neutrophil subsets are mainly classified according to their function and location.^72^ TANs directly affect tumor cell growth, angiogenesis, and distant metastasis by secreting fibroblast growth factor 2, HGF, oncostatin M, neutrophil elastase, matrix metalloproteinase 9, β2 integrin, and vascular endothelial growth factor (VEGF).^73,74^ In addition, C–C motif chemokine ligand (CCL) 2 and CCL-17, derived from TANs, indirectly affect the biological characteristics of liver cancer cells, change the tumor microenvironment, and recruit regulatory T cells (Tregs) and macrophages into liver cancer tissue, which boosts the growth and metastasis of HCC.^75^

A positive feedback loop of tumor stem cell-like cells and TANs in liver cancer is related to tumor progression. In the tumor microenvironment, TANs promote HCC tumor formation, growth, and metastasis by promoting hepatoma cell proliferation, migration, invasion, colony formation, and the negative regulation of antitumor immunity.^76–78^ Tumor cells secrete CXCL5 and promote the infiltration of more TANs to form a positive feedback loop,^78^ and overexpression of PD-L1 in TANs contributes to inhibiting the proliferation and activation of T cells.^79^ TANs can also release NETs to promote the progression of liver cancer,^28^ induce tumor-related thrombosis, aggravate the body’s hypercoagulable state,^80^ and increase the risk of tumor-related complications, such as organ failure.^81^ The tumor growth rate of mice could be slowed down after the deletion of NETs.^28^ In the animal model, deleting TANs improved the therapeutic effect of sorafenib and reduced drug resistance, which provided important evidence for the clinical use of antitumor drugs.^75^

The NLR is an independent marker that can be used to predict the disease-free survival rate at 5 years after liver transplantation^82^ and is also related to the degree of intratumoral macrophage infiltration.^83^ The recruitment of neutrophils in HCC tissues is related to high levels of TGF-β, receptor tyrosine kinase Axl, and CXCL5.^84^ In addition, neutrophils can be recruited by self-secreted IL-6 and IL-8, and these cytokines aggravate tumor cell exudation and matrix angiogenesis.^72^ Neutrophils can also be recruited and polarized by platelets and γδ T cells.^85^ Hypoxia, nutritional deficiency, and cell necrosis within the tumor lead to the release of DAMPs, which further replenish and activate neutrophils.^71^

However, the antitumor effect of neutrophils in the tumor microenvironment should not be ignored. Neutrophils induced by tumor necrosis factor alpha have the capacity to slow tumor growth and metastasis through HGF/MET (MET proto-oncogene, receptor tyrosine kinase)-dependent nitric oxide release.^86^ Meanwhile, TANs can directly kill tumor cells by releasing ROS, stimulating the T-cell response, assisting in antigen presentation, inhibiting early tumor formation, and inhibiting the formation of metastatic foci.^87^ Although this seemingly contradictory conclusion is caused by the complexity of the biological characteristics of neutrophils, more importantly, it reflects the plasticity of the tumor microenvironment.^88^ Once the tumor microenvironment is formed, neutrophils are prone to immunosuppression and adopt a tumor-promoting phenotype.

## Summary

Neutrophil infiltration into the liver is a basic pathological feature of different types of liver disease; however, there are many unanswered issues regarding the kinetics and functions of neutrophils. Neutrophils not only function in anti-infection, immune regulation, tissue repair, and antitumor effects but also cause adverse consequences, including tissue injury, immune paralysis, and promotion of tumor metastasis under the specific tumor microenvironment. In different types and stages of liver diseases, the function of neutrophils may be protective or destructive. Precisely targeting upstream factors or directly affecting the function of neutrophils might become a new direction for the treatment of liver diseases in the future.
